# Impact of competition on microfinance institutions: bibliometric analysis and systematic literature review

**DOI:** 10.1016/j.heliyon.2022.e10749

**Published:** 2022-09-30

**Authors:** Chaerani Nisa, Vive rita, Dony Abdul Chalid

**Affiliations:** a Faculty of Economics and Business, Universitas Indonesia, Indonesia; b Faculty of Economics and Business, Universitas Pancasila, Jakarta, Indonesia

**Keywords:** Microfinance institutions, Systematic literature review, Bibliometric analysis, Competition

## Abstract

Microfinance institutions are challenged by changes in competition, which impact them in various ways. Competition among microfinance institutions is unique because service to the poor, rather than profit alone, is their primary goal. Scientific research on microfinance institutions has increased dramatically over the last two decades. However, previous research lacked a systematic approach in reviewing the literature on the impact of competition in microfinance institutions. This study bridges this gap by reviewing 67 journal articles regarding competition among microfinance institutions. It outlines existing research topics related to the impact of competition on microfinance institutions and offers recommendations for future research. We referred to the RepOrting Standards for Systematic Evidence Syntheses review protocol to conduct a systematic literature review. We also conducted a bibliometric analysis for thematic observations. The results revealed the following four clusters on the impact of competition: social impact, performance, market structure, and relationships with other financial institutions. In conclusion, competition will positively impact microfinance institutions if they accept its inevitability and strive to adapt. Moreover, this study suggests a direction for subsequent research on policies.

## Highlights

• Over 60 articles on competition among MFIs were systematically reviewed.
• ROSES protocol, bibliometrix R package, and thematic analysis were employed.
• Social impact, performance, market structure, and relationships impact competition.
• Competition will positively impact MFIs if they accept its inevitability and adapt

## 1 Introduction

Competition in the financial industry is of great interest because of its persistence following the numerous interactions and trade-offs around stability (S. Li, 2019; Saif-Alyousfi et al., 2018), macro policies (Khan et al., 2016; Segev et al., 2020), and performance (Dai and Guo, 2020; Fang et al., 2019). Many researchers have investigated the impact of competition because of its criticality to welfare-related public policies in the financial sector. They believe that financial deregulation, which motivates competition, encourages capital accumulation and economic progress. Moreover, financial institutions are forced to reduce and increase their lending and deposit rates, respectively, owing to the increased competition in the industry.

The ‘quiet life’ hypothesis proposes that monopoly power ensures the peaceful and competition-free existence of organisations by reaping monopoly rents through slack or inefficiency (Hicks, 1935). Assuming this was the case, lowering the competition level would cause efficiency loss, whereas raising it would exert the reverse impact since management adapts to the challenges. Intense competition motivates the management to improve its performance via cost-saving, diversification, and the provision of improved and fast financial services.

Nevertheless, many factors may restrict or alter the nature of competition and its anticipated consequences in the microfinance market. The conventional view presupposes the existence of competition among profit-maximising businesses (Mirzaei et al., 2013). However, microfinance institutions (MFIs) are unique because most of them acquire external grants and subsidies from non-profit-driven institutions or investors, e.g., non-governmental organisations, social investors, or donors (Barry and Tacneng, 2014). These institutions largely focuon social objectives and prioritise the empowerment of women and the local community. They frequently alternate between lending and training (a labour-intensive activity that does not immediately contribute to production in terms of the loans granted). Therefore, non-profit institutions expend an additional workforce and demonstrate more significant inefficiencies than profit-driven organisations in the business process (Servin et al., 2012). Thus, the resource dependence theory elucidates the financial challenges of non-profit organisations, which are due to dynamic changes in donor priorities, in many low-income countries (Shava, 2021).

Conversely, the agents in non-profit organisations are motivated by non-financial benefits. Thus, value is imposed on matching mission accomplishment (Besley and Ghatak, 2005). The compatibility between the agents and missions of non-profit organisations is manifested in their founder as chief executive officer (CEO) or entrepreneur. The entrepreneur/CEO can achieve dual-bottom-line objectives of an MFI, namely social and financial performances (Randøy et al., 2015). This capacity to aid impoverished people while maintaining financial viability, may explain why the differences in the ownership types or legal status do not cause significant disparities in the performances of MFIs (Tchakoute-Tchuigoua, 2010; Wijesiri, 2016).

Additionally, MFIs grant loans without collaterals to their clients because they generally lack sufficient collaterals (Hermes and Hudon, 2018). Thus, financial institutions require guarantors to avoid asymmetric information issues involving such institutions and their clients. Further, MFIs address this issue by utilising lending technologies, such as dynamic incentives and joint liabilities (Galariotis et al., 2011). However, the emergence of new MFIs can diminish the effectiveness of such technologies (de Quidt et al., 2018a). For example, the presence of new MFIs that offer individual loans rather than joint liabilities reduces the frequency of employing joint liabilities since individual loans do not require the establishment of groups (Guha and Chowdhury, 2013). In a dynamic incentive system, the presence of new MFIs that offer higher loans at cheaper interest rates motivates consumers to take new loans while their existing ones remain active (double-dipping). Concurrently, poor borrowers cannot access loans because of increased monitoring expenses (Hoff and Stiglitz, 1998; McIntosh and Wydick, 2005).

MFIs initially operated as monopolists in the market (McIntosh et al., 2005) and this motivated them into innovating loan technology and management, which enhanced their abilities to serve many people without compromising their healthy financial position (Wright and Rippey, 2003). Significant competition among MFIs began in the late 1990s. Today, microfinance has grown extraordinarily in many emerging markets and developing countries; it has been offering modest loans to the impoverished for the last three decades. There are approximately 139 million microfinance clients globally owing to the rapid growth of MFIs (Haas et al., 2021).

This study demonstrates the uniqueness of MFIs, which impact the competitive landscape differently from what is obtainable with for-profit-only institutions. A detailed systematic literature review on the impact of competition on MFIs was conducted, followed by analyses of the research prospects regarding this topic. MFIs compete with other MFIs and financial institutions. For example, they compete with banks targeting the same client types. The social logic of an MFI as a social entrepreneur produces more affordable microcredit. Comparatively, the market logic of MFIs indicates that they also increase the cost of accessing their loans. However, the presence of additional market competitors may surpass the desire for profit maximisation, thus compelling MFIs to offer more affordable loans (Sun and Liang, 2021). This phenomenon accounts for the emergence of studies on the impact of competition on MFIs and examines the various aspects and perspectives.

Recent studies on competition among MFIs revealed that scholars focused mainly on the relationship between competition and interest rates (Al-Azzam and Parmeter, 2021; Baquero et al., 2018), the ability of MFIs to simultaneously achieve social and financial goals (Hossain et al., 2020; Kar and Bali Swain, 2018b), and the internal strategies of MFIs (Durango et al., 2021). Meanwhile, theoretical contributions demonstrate that studies on competition of MFIs began with observation of one or two MFIs (Navajas et al., 2003; Vogelgesang, 2003) and expanded to include many MFIs (Assefa et al., 2013; Kar and Bali Swain, 2018b). Moreover, these studies generally employed an empirical or theoretical approach to state their conclusions. Only a few have surveyed the existing literature to completely elucidate the competition issues confronting MFIs. Thus, this study bridges the existing research gap by elucidating the impact of competition on MFI via the systematic literature review (SLR) approach. This research also tries to answer how competition affects MFIs by SLR approach. This approach ensured a broadened and diverse perspective on the topic, facilitating an objective identification of the competition impact on MFIsand the predictions of possible issues. Furthermore, it advances the objectives of this study—to conduct a literature review on identifying competition impact on MFIs and predict further research issues.

Additionally, this study employs bibliometric network analysis, which enables researchers to establish the link between writers and identify the common focus of the evaluated articles (Nogueira et al., 2020). Network analysis facilitates the identification of the most significant articles while extracting their outputs and determining their clustering (Bartolini et al., 2019). Further, it elucidates the significant accomplishments along a research direction and the main themes that will attract the attention of the scientific community in the near future (Jes et al., 2021). Therefore, we anticipate that the utilisation of bibliometric network analysis will help us achieve the aims of this study to investigate the impact of competition on MFIs and identify research gaps to generate future research topics.

The existing SLR of MFIs focused on Islamic MFIs (IMFIs) (Mohamed and Fauziyyah, 2020), the evolution of MFIs (Gutiérrez-Nieto and Serrano-Cinca, 2019; Nogueira et al., 2020), and the governance of MFIs (Rasel and Win, 2020). This study is motivated by a desire to comprehensively elucidate how competition impacts MFIs. These insights are crucial to successfully foster economic development and poverty eradication and pursue commercial goals. This review offers the following contributions to the literature: first, it systematically assesses works that significantly relate to the research topic, thereby generating clusters via bibliometric analysis. Second, it employs thematic map analysis to help academics and policymakers identify research gaps and recommends future initiatives. The findings of this study provide information that can help MFIs confront competition. Practitioners and academics can rely on this study to identify the general impact of competition from diverse perspectives. The result have managerial implications on how to respond when MFIs deal with competition; therefore, it may benefit MFIs. Numerous studies on competition have been conducted from various perspectives and have achieved mixed results.

Notably, this study is among the first to conduct SLR of previous related research to establish thematic clustering. Therefore, it summarises the overall findings of the numerous research, and establishes the common trend by arranging the findings in the form of clusters. This method is beneficial to inform and categorise the impact of competition on MFIs. The findings identified four clusters of competition's impact on MFIs: social impact, performance, market structure, and interactions with other financial institutions. Thus, if MFIs recognise the necessity of competition and work to adapt, competition will benefit them. Additionally, this study shows the way forward for future policy research.

The remainder of this paper is structur'd, as follows. Section 2 discusses the methods of the study, Section 3 highlights the findings of the bibliometric and content analyses of the selected papers related to the impact of competition on MFIs. Section 4 describes the future research direction based on thematic analysis and Section 5 presents the conclusions.

## 2 Methods

SLR differs from traditional literature reviews by adopting an organised, transparent, and replicable approach to minimise bias in deciding the articles to be included or excluded from the review (Greyson et al., 2019). Furthermore, it emphasises the evaluation and synthesis of the available materials. Rijal et al. (2022) define SLR as a comprehensive and well-organised literature study, backed by a sound methodology that is led by the central research questions. SLR also guarantees an accurate search strategy that leads to a reliable and transparent result. However, we did not identify an SLR method that was specially designed for financial institutions. Durach et al. (2017) proposed methodological guidelines, which was particularly based on supply chain management. Contrarily, Linnenluecke et al. (2020) noted that authors could tailor the SLR approach to meet their specific goals since the method is not standardised. Therefore, we used the approaches of Chakraborty et al. (2021), Linnenluecke et al. (2020), and Mohamed Shaffril et al. (2020) as guidelines for our SLR and bibliometric analyses.

The RepOrting Standards for Systematic Evidence Synthesis (ROSES) is a method to synthesise systematic evidence, including systematic reviews (Haddaway et al., 2018). Shaffril et al. (2020) employed ROSES as a guide for conducting SLR. Additionally, bibliometric analysis is a method for visualising data and thoroughly analysing an existing theme (Chakraborty et al., 2021; Linnenluecke et al., 2020). We thoroughly analysed the impact of competition on MFIs using both methods.

### 2.1 Review Protocol–ROSES

Haddaway et al. (2018) discussed the benefits of ROSES, including ensuring a transparent procedure during a systematic review. Additionally, although Haddaway et al. (2018) developed ROSES for environmental studies, they emphasised its suitability for diverse studies. Shaffril et al. (2021) employed ROSES throughout the research stage, which comprised the definition of their research objectives and the development of their systematic search strategy, including identification, screening (determination of the inclusion and exclusion criteria), and eligibility. The subsequent stage involved quality appraisal, where further checks were conducted to ensure that the selected articles satisfied the criteria. The final stage explained how the data were gathered for review, processed, and validated. We referred to Shaffril et al. (2021) in implementing ROSES in our research.

### 2.2 Research question

Following Shaffril et al. (2020), this study employed the Population, Interest, Context (PICo) format, which is a tool to establish a suitable research question. Here, the population, interest, and context correspond to MFIs, the impact of competition, and global evidence, respectively. Thus, the main research question is: What is the impact of competition on MFIs? This primary research question will lead to the second one: What is the research gap regarding this topic that may lead to a potential research direction?

### 2.3 Systematic search strategies

The subsequent step was to implement systematic search procedures comprising the following three stages: identification, screening, and eligibility.

#### 2.3.1 Identification

This study employed Scopus as the primary database to search for article sources. Further, Google Scholar and ScienceDirect accounted for additional databases.. As we utilised Scopus as the only primary database, the entire data could not be collected. There was a risk that data from other databases, such as the Web of Science, would be omitted. However, Scopus is a database with much research in social sciences, and it exhibits an easy-to-utilise interface (Pérez-Gutiérrez and Cobo-Corrales, 2020). Thus, we asserted that we had gathered sufficient data to sketch the academic thinking, research hubs, and other analyses undertaken in this study. Meanwhile, ScienceDirect is widely used by other SLR studies such as Chakraborty et al. (2021) and (Wu et al. (2020). Following Adams et al. (2016), Google Scholar results fluctuate at an incredibly rapid rate with a varied propensity for results to be higher or lower than in a previous search as new content is added over time; the results vary at a swift pace – sometimes in only a matter of hours. Thus, we follow Gutiérrez-Nieto and Serrano-Cinca (2019) and utilised only the findings on the first page since Google Scholar organizes its results according to their relevancy. The main keywords employed in this study were microfinance and competition. In addition to the two main keywords, the identification also employed synonyms obtained from Scopus or previous research. Table 1 summarises the search strings employed by Scopus, ScienceDirect, and Google Scholar. The search conducted during18–25 October 2021 yielded 190 articles (159, 21, and 10 articles from Scopus, ScienceDirect, and Google Scholar, respectively).

**Table 1:** Selection strategy.

Database	Search String
Scopus and ScienceDirect	TITLE-ABS-KEY ((competition OR “market structure”) AND (microfinance OR microbank∗ OR microcredit∗))
Google Scholar	((competition OR “market structure”) AND (microfinance OR microbank∗ OR microcredit∗))

#### 2.3.2 Screening

There are few articles that are available in more than one database. The authors removed the duplicated articles, which resulted in 165 articles. The following stage involved screening the articles. Screening refers to the criteria by which an author included or excluded articles from the analyses. For example, an author can utilise time (Shaffril et al., 2020) or publication type as screening criteria. This study was not limited to time; it focused on peer-reviewed journal articles to ensure their quality. Only articles that were written in English were selected. Table 2 presents the inclusion and exclusion criteria.

**Table 2:** Inclusion and exclusion criteria.

Inclusion	Exclusion
English article	Non-English article
Article from a peer-review journal	Article from a conference or a book

#### 2.3.3 Eligibility

In this stage, we conducted manual checks by evaluating the titles and abstracts to ensure that all articles satisfied the desired criteria. Several articles, which were related to competition but did not explain its impact on MFIs, were excluded from the list. Following the eligibility check, we obtained 69 articles.

### 2.4 Quality assessment

**Figure 1:**
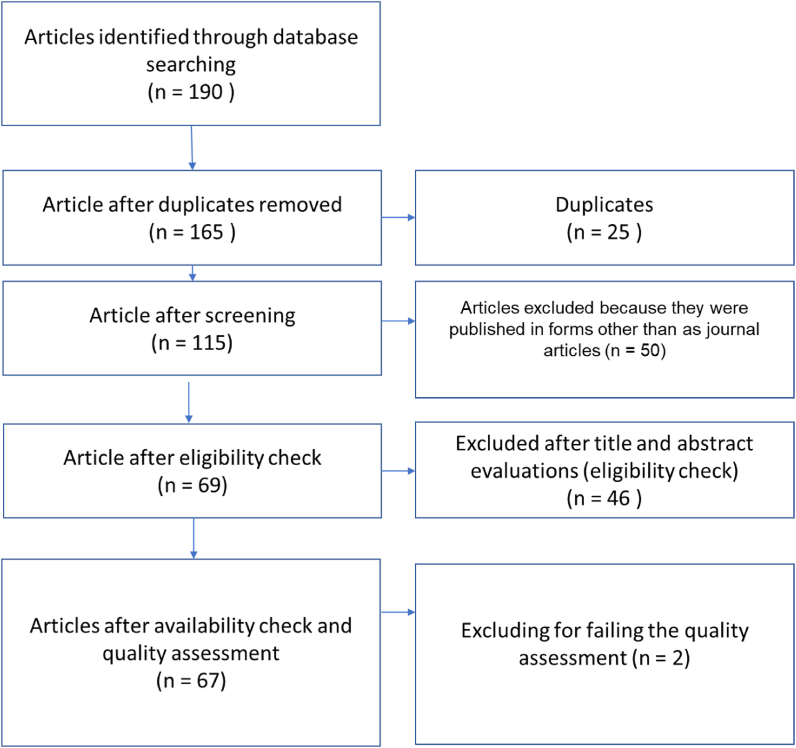
Article selection process.

This stage involved the validation process for the retrieved articles. Tranfield et al. (2003) stated that there were no optimal tools for assessing articles; this study referred to Rasel and Win (2020), who selected papers from publications that were indexed by Scopus, Web of Science, or both. However, we utilised Scopus as our primary tool to validate the retrieved articles. We chose articles that were listed in the Scopus website during the specified period. Accordingly, this study examined a total of 67 articles. Figure 1 shows the article selection process.

### 2.5 Data presentation and analysis

Following Hassan et al. (2021) and Nogueira et al. (2020), we conducted a descriptive analysis by displaying information on the development of articles by examining the journals, authors, and countries. Furthermore, we performed the clustering via network and content analyses by examining co-citations and co-occurrences via bibliometric analysis. Bibliometric analysis was performed employing the R software and VOSviewer. In addition, we conducted thematic analysis by examining the pattern similarity of the entire article.

#### 2.5.1 Descriptive and network analyses

Numerous studies have motivated further research to elucidate the previous findings. Bibliometric analysis is a method to describe, evaluate, and monitor existing research (Kumar et al., 2019) and entails objective and reliable explanations (Aria and Cuccurullo, 2017). Aria and Cuccurullo (2017) divided the bibliometric technique into citation analysis, co-occurrence, and co-authorship. Citation analysis focuses on citations to estimate closeness between journals, articles, or authors, and it consists of bibliographic coupling and co-citation analysis. Co-occurrence technique employs keywords or important words to develop similarity while co-authorship denotes the contribution of two or more writers or organisations as analysis tools.

This study employed the following two commonly utilised software packages to conduct bibliometric analysis-type SLRs: bibliometrix (bibliometric analysis based on the R statistical programming language) and VOSviewer. These two programmes are mutually beneficial. We utilised bibliometrix for the R program, which was created by Aria and Cuccurullo (2017) to elucidate the descriptive analysis and research development of the competitiveness of MFIs. Moreover, we employed VOSviewer to better comprehend the relationships between the articles and build the clusters because it enabled data cleansing via the thesaurus file. Thesaurus can combine synonyms or change author names and journals, thereby allowing for a more accurate thematic analysis via clustering (Gutiérrez-Nieto and Serrano-Cinca, 2019). The descriptive analysis elucidated the article characteristics that discussed the impact of competition on MFI. The descriptive analysis revealed the most significant articles and journals, their period and development, and the authors’ countries of origin. We analysed all the articles for further in-depth observations. Thus, descriptive analysis facilitated the preparation of data for further analysis (Chakraborty et al., 2021).

## 3 Results

### 3.1 Descriptive analysis

Table 3 summarises all the articles. The data indicated that the literature on competition among MFIs started accumulating in the early 2000s. This result is consistent with the assertion of Wright and Rippey (2003) that MFIs initially operated as a monopoly before transitioning into a competitive market in the late 1990s.

**Table 3:** Fundamental facts regarding the source of the data.

Criteria	Description	Results
Main information	Period	2001–2021
	Sources (journals)	50
	Documents	67
	Average publication per year	6.7
	Average citations per document	20.67
	Average citations per year per documents	2.224
	References	2839
Document types	Articles	67
Document contents	Keywords Plus (ID)	114
	Authors' keywords (DE)	174
Authors	Authors	130
	Author appearances	145
	Authors of single-authored documents	20
	Authors of multi-authored documents	110
Authors Collaboration	Single-authored documents	20
	Documents per author	0.515
	Authors per document	1.94
	Co-authors per documents	2.16
	Collaboration index	2.34

In total, 67 papers were retrieved from 50 journals, indicating that only a few journals were dominant. As Table 4 describes only 8 out of 50 journals that contributed more than 1 article. The average annual number of journal articles was 6.7, with 20 of the 143 authors being sole authors. The first article was published in 2001 by Hollis and Sweetman (2001), while the last article was published in 2021 by Deb and Sinha (2021). The former described the competitiveness of an MFI in Ireland, whereas the latter examined the competition confronting several MFIs. This condition also described the dynamics of the articles on the competitiveness of MFIs. Many authors had initially observed one or two MFIs, and the articles later expanded to include the MFIs of an entire country, as well as those globally.

**Table 4:** Rankings of the most productive and influential journal.

	Articles	Percentage of total local sources	Total citation	Number of articles cited by local sources	h-index	g-index	M-index	Total number of citations per article	First Article
Journal of Development Economics	4	6%	234	160	4	4	0.24	58.5	2005
Journal of International Development	3	4.5%	78	29	3	3	0.15	26	2003
Small Enterprise Development	3	4.5%	78	6	3	3	0.15	26	2002
European Journal of Development Research	2	3%	8	3	2	2	0.5	4	2018
International Journal of Social Economics	2	3%	8	6	2	2	0.25	4	2014
Applied Economics	2	3%	83	43	2	2	0.22	41.5	2013
Journal of Banking and Finance	2	3%	294	89	2	2	0.15	147	2009
World Development	2	3%	127	131	2	2	0.11	63.5	2003
Empirical Economics	1	1.5%	3	6	1	1	1	3	2021
International Journal of Finance and Economics	1	1.5%	1	3	1	1	1	1	2021

Note: *Percentage of total local source,* articles divided by local source (67 articles); *Total number of citations per article,* Total citations divided by articles.

Table 4 lists the most productive journals in the data source. The *Journal of Development Economics* (JDE) contributed the most and accounted for four articles followed by the *Journals of International Development* and *Small Enterprise Development* with three articles each. JDE was also the most-cited journal by local sources. Local source refer to 67 articles that we analyse in this study; 160 articles in JDE are cited by local sources. JDE was followed by World Development, with 131 articles, which were cited by local sources. The Journal of Banking and Finance was the next most influential journal, with 89 articles, which were cited by local sources.

The source impacts were measured by the h-index, M-index, and g-index. h-index refers to the number of publications that are attributed to a scientist, as well as the influence of those publications on the scientist's peers (Hirsch, 2005). Although the g-index utilises the same ranking of a publication set as the h-index, it refers to the highest number of papers that obtained g^2^ or more citations together (Bornmann and Daniel, 2008). The g-index is calculated by ranking articles in decreasing order of the number of citations; the g-index is the highest unique number such that the top g articles received at least g^2^ citations collectively. Similar to g-index, h-index is calculated by ranking articles based on the number of citations, h equals the number of papers (N) in the list that have at least N citations. M-index is calculated by dividing a journal's h-index by the number of years since its first cited publication. JDE scored the highest h- and g-indexes, indicating that all its published articles exerted significant impact. Regarding the m-index, which calculates the dynamics of the h-index, JDE scored 0.25. This lower m-index was because they had been publishing competition-related topics since 2005. Concurrently, since Empirical Economics and the International Journal of Finance and Economics published their first articles, which had been cited, in 2021, they scored higher m-indexes.

**Figure 2:**
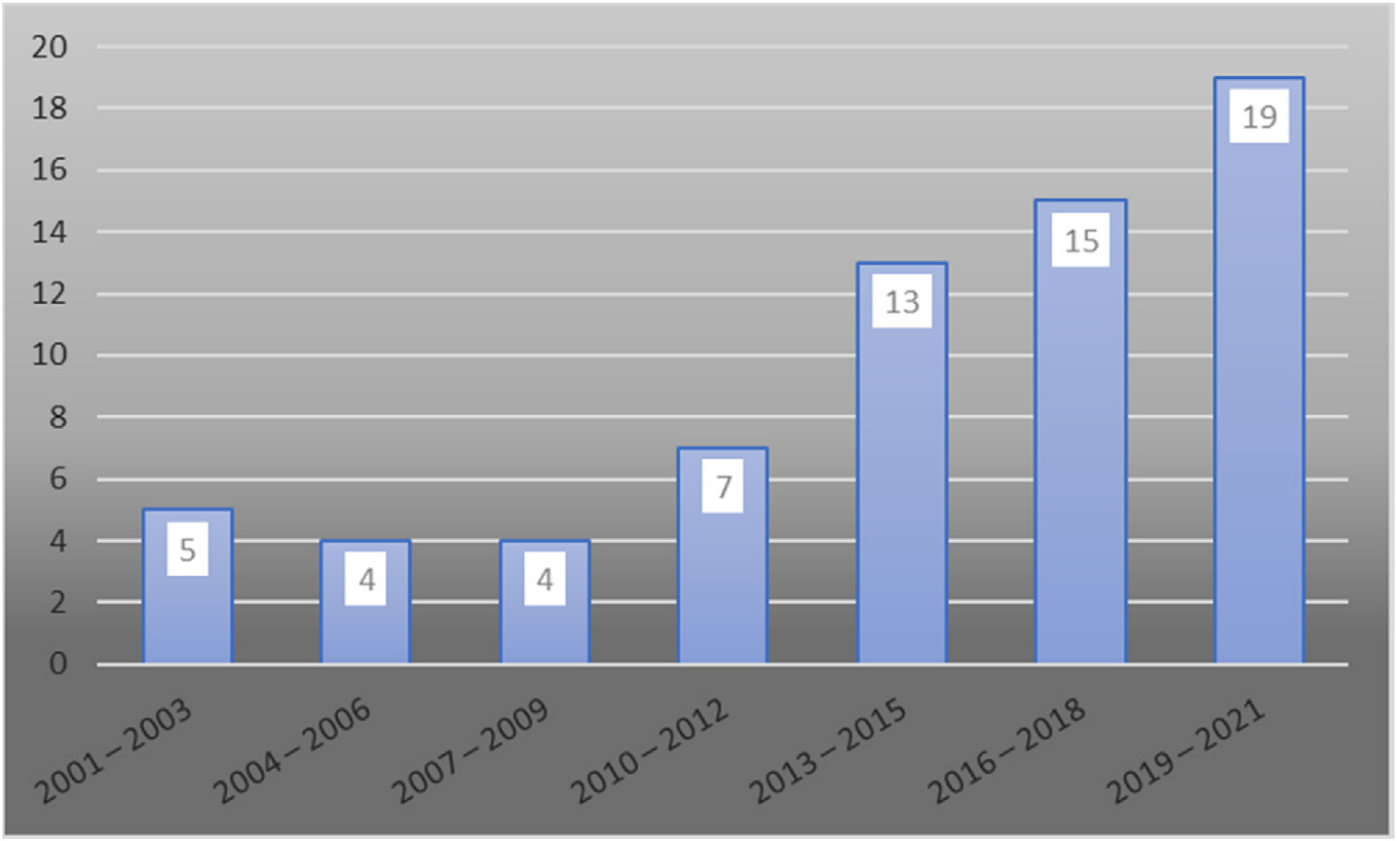
Publication development regarding the competitions among MFI.

Figure 2 shows the annual number of scientific publications, as well as the increasing trend in publications, with a significant increase in 2013. A major reason for this increase was the Andhra Pradesh incident in 2010, where stiff competition among MFIs contributed to the microfinance crisis (Wondirad, 2020). This incident attracted the attention of many academics and yielded many articles that were related to competition among MFIs.

The most influential articles based on citations by local sources are listed in Table 5. Following Linnenluecke et al. (2020) and X. Xu et al. (2018), we develop top cited articles by local source. The most cited article by local sources and the most influential on competition is that of McIntosh and Wydick, 2005. Concurrently, Mersland and Strøm (2009) attracted the most citations among all the related articles globally. Nevertheless, McIntosh and Wydick, 2005 strongly emphasised the competition among MFIs from a theoretical approach, making it is a cornerstone of other publications. The second most cited article was that of Assefa et al. (2013), which was published in the then *Journal of Applied Finance Economics* that was changed in 2014 to *Applied Economics*. This journal was not included in the Scopus Q1 journal. However, Assefa et al.' (2013) were the first to apply the competition estimate of banks to MFIs; thus, they attracted many citations.

**Table 5:** Top ten most influential articles based on local citations.

Document	Local Citations (LC)	Global Citations (GC)	LC/GC Ratio (%)
McIntosh and Wydick, 2005*Journal of Development Economics* 78: 271–298	29	177	16.38
Assefa et al. (2013)*Applied Financial Economics* 23 (9) 767–782	16	89	17.98
Navajas et al. (2003)*Journal of International Development* 15 (6) 747–770	12	60	20.00
Vogelgesang (2003)*World Development* 31 (12) 2085–2114	12	87	13.79
Kar (2016)*Review of Applied Economics* 30 (4) 423–440	7	12	58.33
Mersland and Strøm (2009)*Journal of Banking and Finance* 33 (4) 662–669	7	284	2.46
Vanroose and D'Espallier (2013)*Applied Economics* 45 (15) 1965–1982	6	80	7.50
Guha and Chowdhury (2013)*Journal of Development Economics* 105 86–102	5	28	17.86
Kar and Bali Swain (2018a)*Applied Economics* 50 (1) 1–14	3	3	100.00
Ghosh and Van Tassel (2011)*Economics Letters* 112 (2) 168–170	3	27	11.11

Note: *LC/GC Ratio,* local citations divided by global citations.

**Figure 3:**
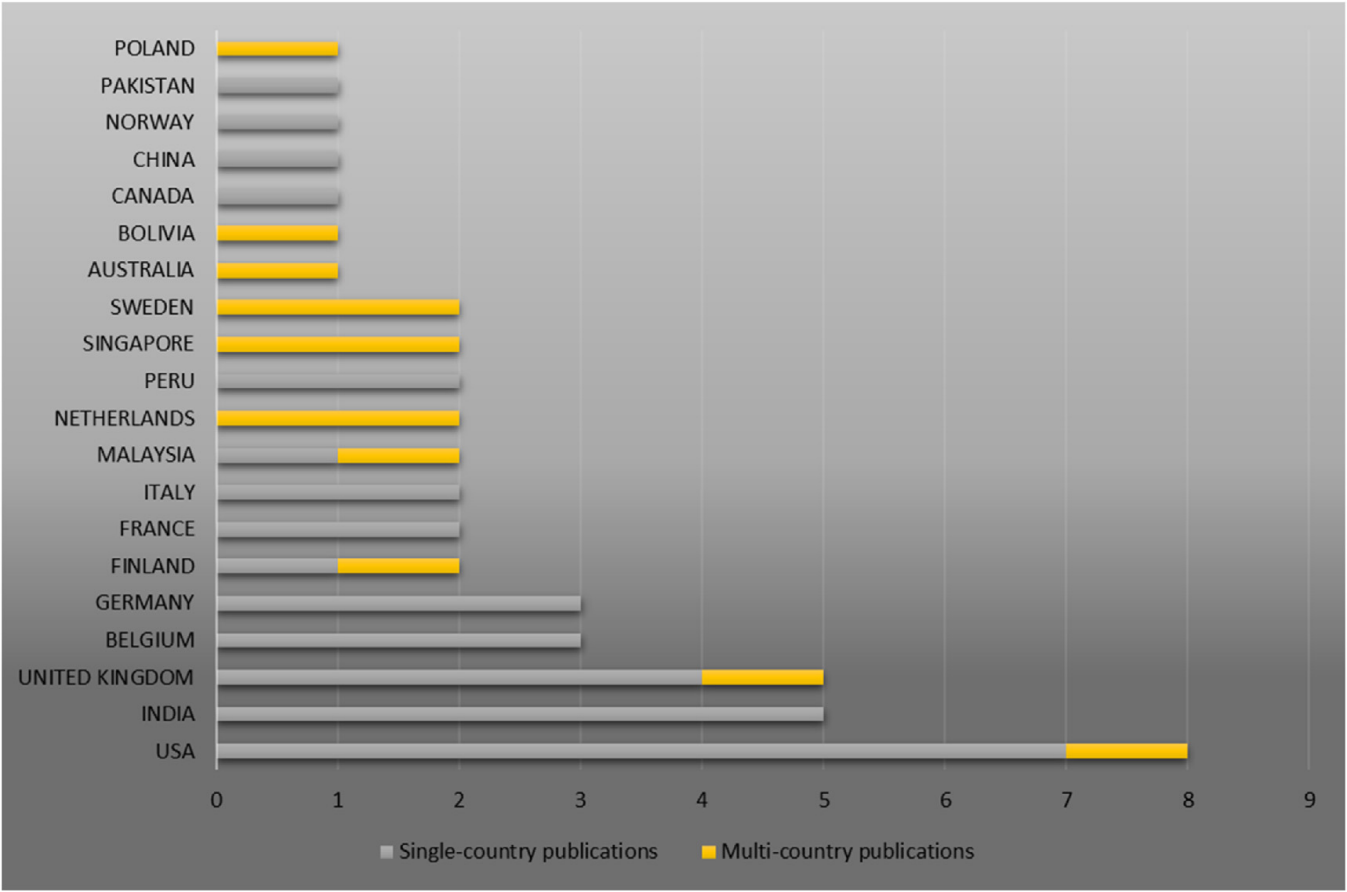
Corresponding authors' countries.

Figure 3 shows the authors’ country of origin. The United States accounted for the most authors, followed by India and the United Kingdom. All the publications from Indian authors were classified as single-country publications. This classification is consistent with the circumstance in India, which accounts for one of the highest numbers of MFIs worldwide.

### 3.2 Network analysis

The visualisation of bibliometric data is inextricably linked to the generation of a network that describes the interaction among publications. Thus, network analysis is generally employed to visualise the data derived via bibliometric analysis and subsequently expressed as a network. Co-citation, bibliographic coupling, and co-occurrence were employed to demonstrate the bibliometric network in this study. We utilise VOSviewer version 1.6.17 to conduct co-citation, bibliographic coupling and co-occurrence analysis.

#### 3.2.1 Co-citation analysis

**Figure 4:**
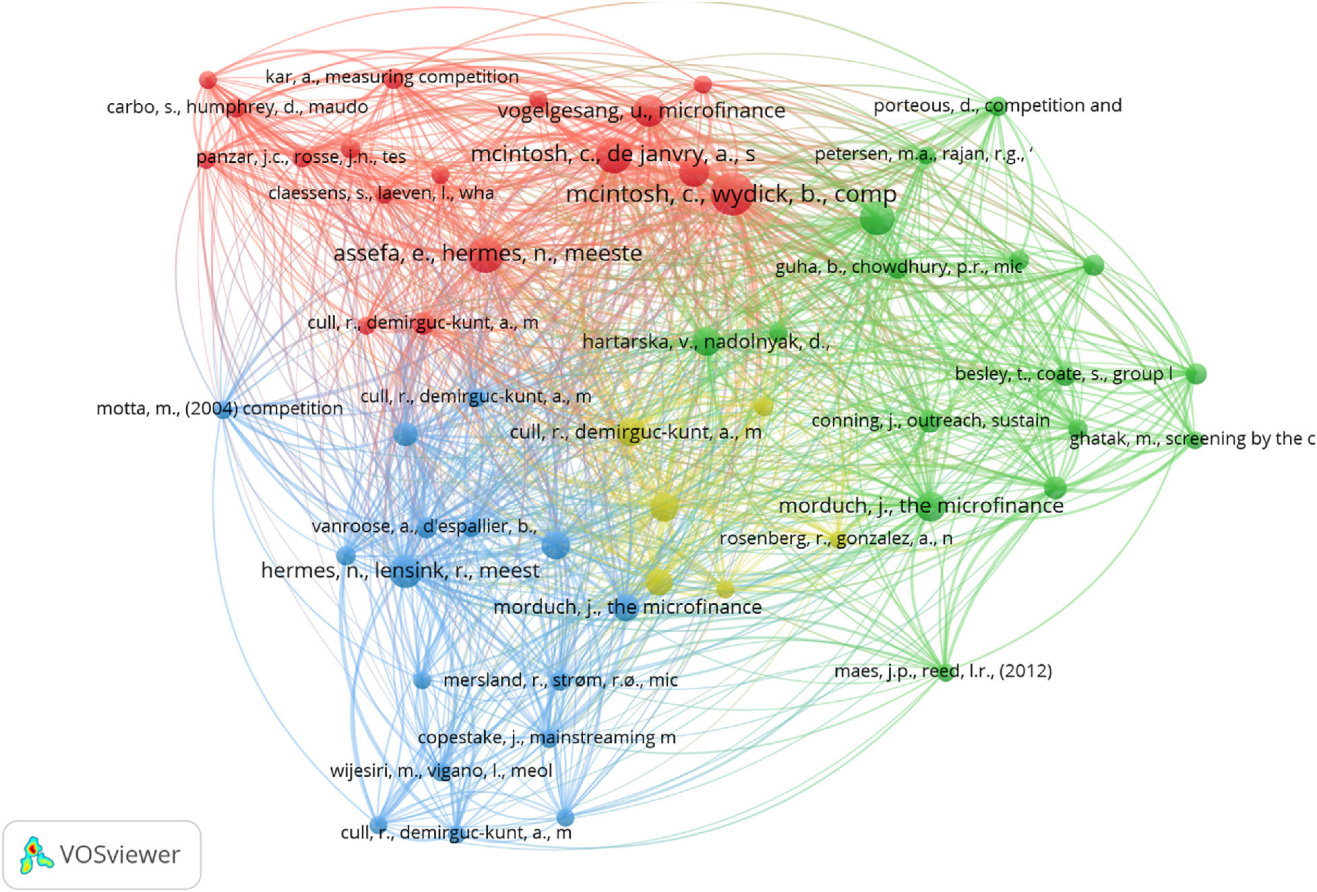
Co-citation among publications.

Figure 4 shows the co-citations of the articles. Two articles are linked through co-citation if a third article cites the two of them. If additional articles cite the two articles, it would strengthen their relationship. Concurrently, the larger the co-citation circle, the more citations an article would receive. The distance between two articles describes the association between journals, particularly the number of journals that cite both publications simultaneously. McIntosh and Wydick, 2005 and McIntosh et al. (2005) exhibited the highest total link strength, indicating that the two papers were frequently co-cited. In co-citation analysis, four clusters were formed, each representing a different theme. In every cluster, there is a topic similarity between every paper. The red, blue, green, and yellow clusters correspond to performance, social issues in general, the market structure of MFIs, and the interactions of MFIs with other institutions, respectively.

#### 3.2.2 Bibliographic coupling

**Figure 5:**
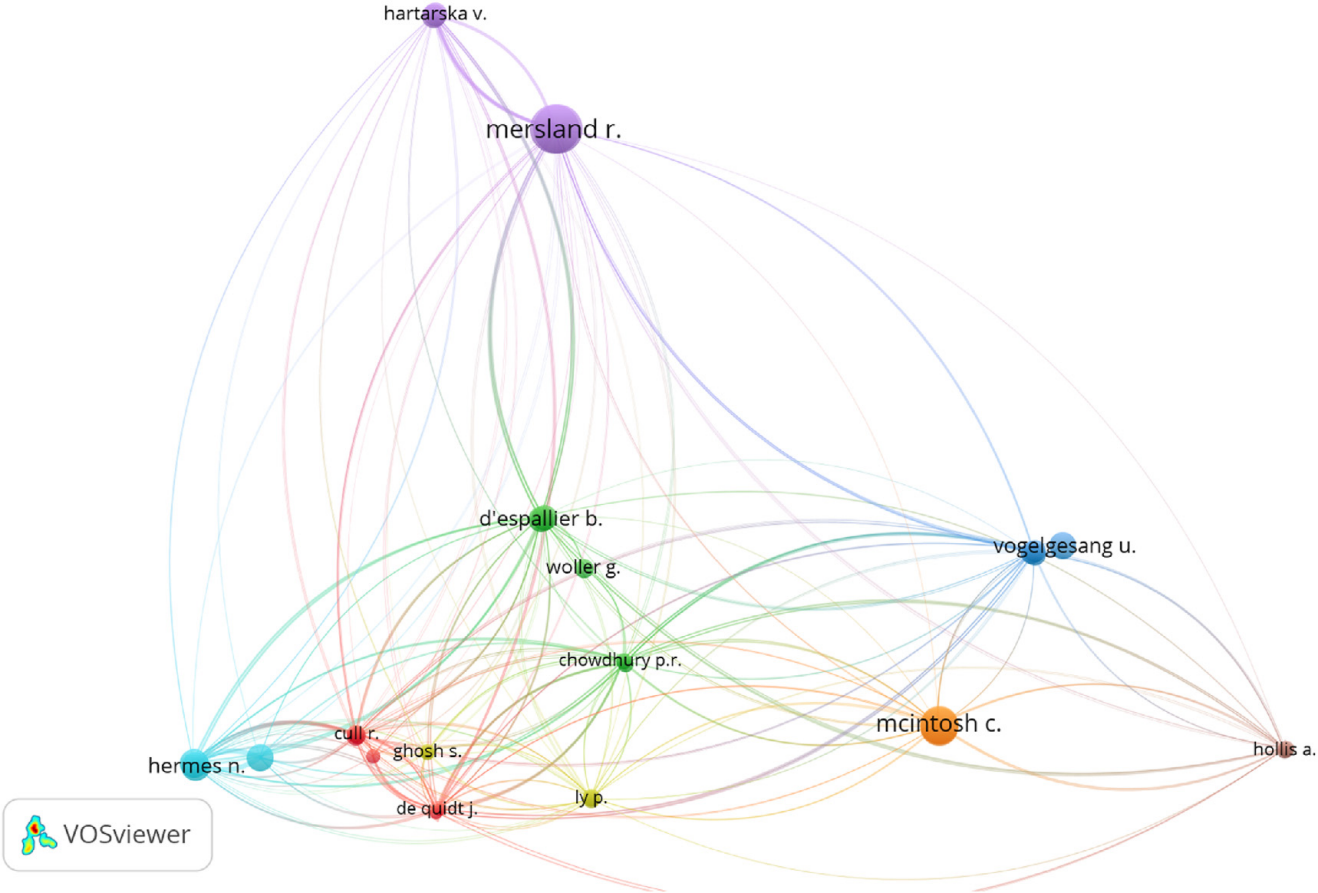
Bibliographic coupling by authors.

Figure 5 shows the bibliographic coupling involving all the studied publications by examining their authors. To perform the analysis, bibliographic coupling was limited to authors of publications with a minimum of 20 citations. This was necessary to obtain relevant clusters via VOSviewer cluster visualisation (Hassan et al., 2021). Thus, 30 authors were identified via the filtering process. In bibliographic coupling, relatively close authors cite the same publication, while distant ones cite different publications.

Figure 5 shows the result of this series of procedures. It is divided into eight clusters. Conversely, the four most significant clusters exhibited almost the same image as the cluster picture based on co-citation. Figure 5 also reveals that some writers from the brown and purple clusters exhibited a minor link, indicating that the two authors did not share references. Meanwhile, the author of the green cluster is close to those of the five other clusters. Meesters, Hermes, de Quidt, and Ghatak published two articles, with the four works sharing the most references. This indicates that the four articles represented the vast majority of the other works.

**Figure 6:**
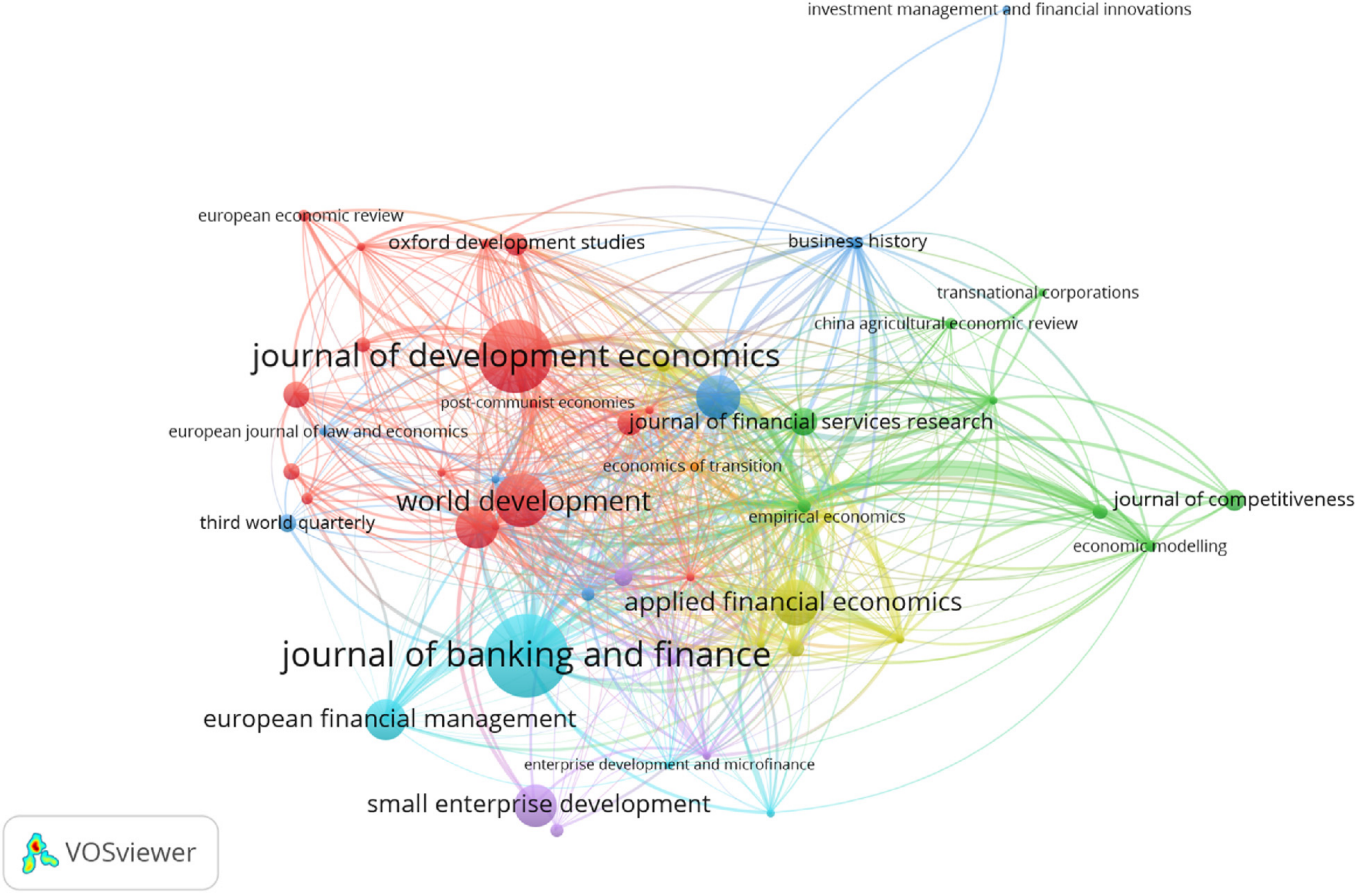
Bibliographic coupling by source.

Figure 6 shows the bibliographic coupling of 50 journal sources. Two journals were eliminated from the list because they lacked bibliographic connections with the others. Seven clusters were formed via these steps (Figure 6). The red cluster was strongly connected to the other six clusters, indicating that it had the most similar references to those of the other clusters. This cluster was dominated by the social impact of competition, with one of the primary concerns of the impact of competition on MFIs being the diminished social capacity.

#### 3.2.3 Co-occurrence analysis

**Figure 7:**
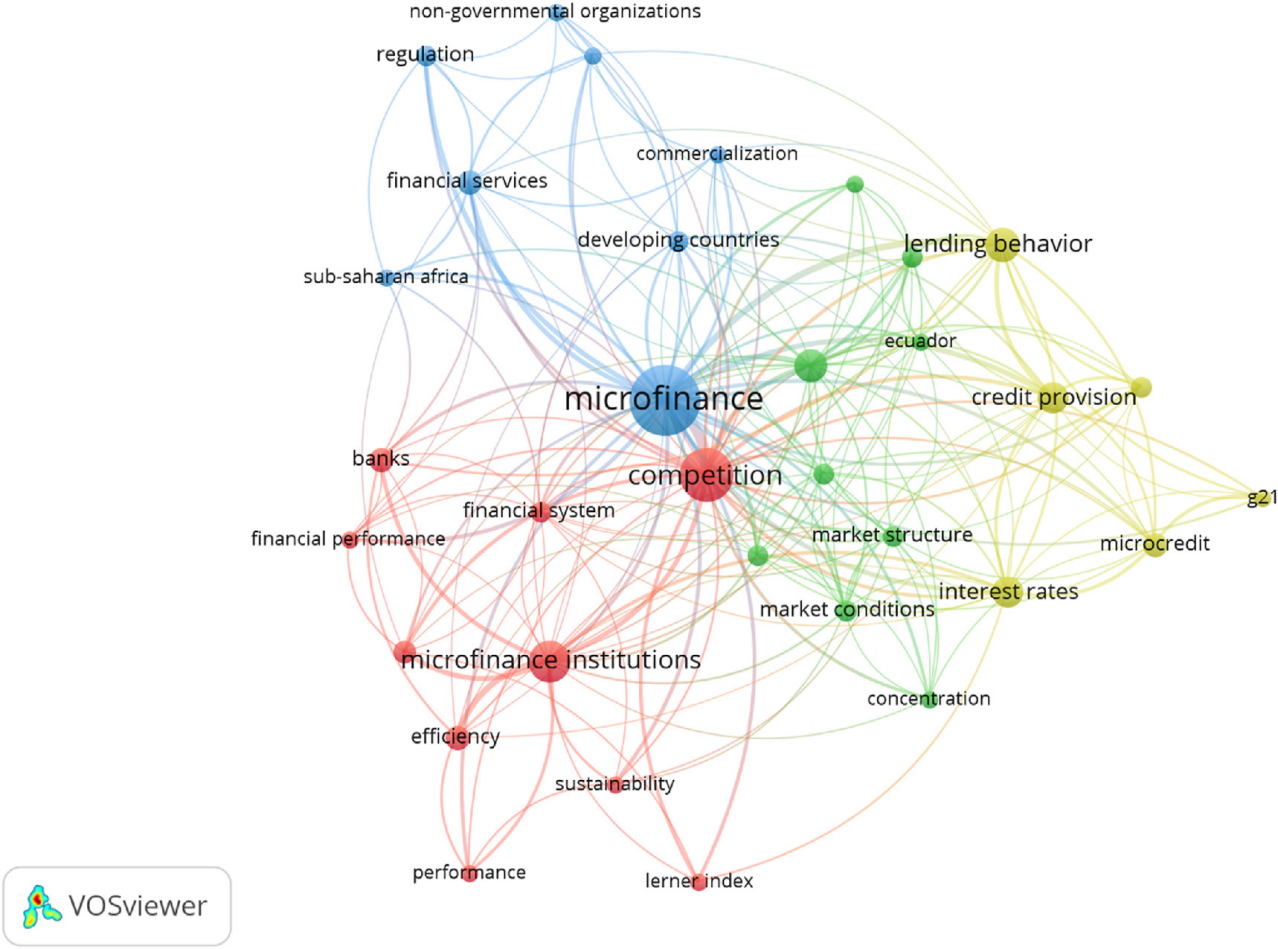
Co-occurrence of keywords.

We then examined the co-occurrence of the utilized keywords used to elucidate the findings, as shown in Figure 7. Co-occurrence analysis was performed on all the keywords, including the author and index keywords. Subsequently, we established a threshold, presumably three or more appearances of a keyword. Thus, we identified five groups via the filtering process and added a minimum of two keywords to each cluster to further narrow the investigation.

Following the competition, the MFI node dominated the red cluster, with most terms relating to the competition between MFIs and other financial institutions; the microfinance node dominated the blue cluster. This cluster was dominated by discussions about developing countries and their regulations. We categorized the blue group as the societal outcome of competitions among MFIs. The discussed topics in the green and yellow clusters were similar. Upon further examination, we concluded that the yellow and green clusters emphasized the performance or activity of MFIs and the structure of the MFI market, respectively.

### 3.3 Thematic observations: impact of competition on MFIs

In this stage, we utilize findings in network analysis to categorise the theme into four clusters.

#### 3.3.1 Cluster 1: social aspect

MFIs have become an attractive research topic for academics because their existence is expected to alleviate global poverty. Thus, when confronted with competition issues, there are concerns regarding whether MFIs can continue to deliver their social responsibilities (Woller, 2002), which may involve reaching out to the disadvantaged (Kai, 2009; Mukherjee, 2014; Olsen, 2010) or empowering women (Deb, 2020). Kai (2009) and Aoun et al. (2019) confirmed that competition limits MFIs from reaching out to the poorest communities. However, Deb (2020) argued that macroeconomic variables, rather than competition, affect the social reach capacity of MFIs. Another viewpoint noted that competition did not contribute to the neglected status of the poor, and attributed it to the flaws in subsidy distribution (Mukherjee, 2014) and a lack of collaboration among MFIs (Zeller, 2006).

MFIs with a social mission suffer the consequences of losses due to competition (Guha and Chowdhury, 2013; McIntosh and Wydick, 2005) because competition can generate asymmetric information on MFI clients. However, under certain circumstances, the lender or borrower may benefit from such a situation (Guha and Chowdhury, 2014). de Quidt and Ghatak (2018) explored the competitive approach of for-profit MFIs and emphasised the need-to-know market power while designing welfare-enhancing policies. Along with the targeted borrowers, the most effective lending technology for the poor also constitutes an issue (Ahlin and Waters, 2016; de Quidt et al., 2018b) because this technology frequently fails in impoverished areas when the business is competitive.

Country-specific studies were also included in some observations on competition and the social aspects (Ashta et al., 2015; Hollis and Sweetman, 2001; Mia et al., 2019). According to the three cited studies, competition facilitates the deterioration of services to the poor. However, Navajas et al. (2003) observed that differentiation in servicing the poor could help MFIs survive competition in Bolivia. Afonso et al. (2016) proposed that MFIs could expand their social impact by serving the much poorer communities. This technique has been effectively employed in China (Xiang et al., 2014). Political issues constituted a critical consideration in assessing the impact of competition on the social capabilities of MFIs in another study (Bédécarrats et al., 2012). Muhammad (2010) noted that competition was due to the growth of the industry in Pakistan.

#### 3.3.2 Cluster 2: competition and the performance of MFIs

In addition to the impact of competition on the outreach capacity of MFIs, many authors have also considered their performance. Performance can be appraised in terms of efficiency (Hartarska and Mersland, 2012; Mersland and Strøm, 2009), financial sustainability (Assefa et al., 2013; Vogelgesang, 2003), and interest-rate stability (Al-Azzam and Parmeter, 2021). Hossain et al. (2020) confirmed that competition exerted a detrimental effect on performance. However, Kar and Bali Swain (2018b) reported the contrary. Thus, there were contradictory empirical evidence on the direct effect of competition on sustainability. Similar conditions were observed in terms of efficiency performance. Growing competition and regulation have eroded the capacity of MFIs to strike a balance between assisting the poor and operating efficiently (Hussain et al., 2020; Mia et al., 2018). This is particularly true of MFIs with social objectives. Moreover, Deb and Sinha (2021) concluded that competition improved the efficiencies of MFIs. These efficiency gains further incentivise MFIs to reduce their interest rates (Al-Azzam and Parmeter, 2021). This scenario is most prevalent in for-profit MFIs (Baquero et al., 2018), where interest rates are sensitive to the changes in the competition.

Concurrently, regarding customer experience, competition does not play any significant role in determining the success of MFIs (Rashem and Abdullah, 2018). By redefining over-indebtedness, Schicks (2013) argued that over-indebtedness must not be blamed on competition because competition offers borrowers many benefits. The competition that is addressed by the deployment of modern technology helps improve the performance of MFIs (Y. L. Li et al., 2019), and lower their interest rates and borrowers’ default (Durango et al., 2021), thereby allowing MFIs to service a variety of clients. According to Chen and Weiss (2007), a shift in the goals of MFIs toward small enterprises is a solution to overcome competition issues since small businesses are essential to the economy.

Affordable interest rates while still supporting the viability of MFIs are critical. However, Caballero-Montes et al. (2021) reported that the interest cap policy is ineffective to overcome heavy competition. Another method to mitigate the adverse effects of competition is to differentiate between credits contracts (Casini, 2015) or develop new products (Sander, 2004). The presence of a new MFI that is more adaptable to a competitive environment can also cause various concerns (Ayayi and Wijesiri, 2018).

Competition related to MFI performance regarding funding is examined in the literature (Ly and Mason, 2012; Ullah et al., 2019). Ly and Mason, 2012 reported that competition hinders the ability of MFIs to secure donor subsidies even though it can increase their efficiency (Ghosh and Van Tassel, 2011). Conversely, Ullah et al. (2019) stated that subsidies and grants, which are subject to particular restrictions, could motivate MFIs to reach out to the poor regardless of the competition level.

#### 3.3.3 Cluster 3: market structure of MFIs

MFI researchers generally attempt to elucidate the structure of the MFI market as a method to determine the competition level. Although whether the market structure represents competition is arguable (Bikker and Haaf, 2002), a concentrated structure also describes low competition in the case of MFIs (Navin and Sinha, 2019). Understanding the market structure is inextricably linked to the need to establish proper MFI regulations. The market structure is generally classified as a monopoly, monopolistic, or perfect competition (Kar and Bali Swain, 2018a). Each country's MFI market structure is unique and varies annually in certain cases (Kar, 2016; Kar and Bali Swain, 2018a; Mia, 2018; Sabi, 2013). These variations are due to MFI mergers or regulator-imposed rules.

The influence of the market structure would be investigated in following research. A competitive market structure encourages the independence of MFIs and their increased assistance of the poorest (Gueyié and Fischer, 2009). However, Pati (2019) opined that a large market share can help MFIs fulfill their social commitments. Another study posited that a competitive MFI market structure would make it challenging for the small business sector to access financial services (Sonnekalb, 2014). This finding is consistent with the information-based theory, which states that increased competition diminishes relationship lending with opaque businesses. This conclusion is congruent with that of Becchetti and Pisani (2010) who concluded that borrowers benefit more in monopolistic and zero-profit market settings.

Many authors consider how the market structure impacts regulation efficiency. Regulations aimed at lowering credit risk can be effectively applied under minimum competition (Karimu et al., 2019). Contrarily, such regulation becomes ineffective when the competition level is high. Thus, regulators must consider the risks that must be regulated. Aguilar and Portilla (2020) adopted a different approach to analysing market power by investigating the influencing elements. The efficient structure hypothesis was proven based on their findings on MFIs in Peru.

#### 3.3.4 Cluster 4: relation with other financial institutions

Historically, MFIs emerged because the financial industry had not yet reached all sectors of the society, particularly the poor (Vanroose and D'Espallier, 2013). The second frequently asked question is whether MFIs are still required considering the expansion of the financial industry. According to Vanroose and D'Espallier (2013), the substitution condition occurs when the development of other financial institutions reduces the social outreach or sustainability of MFIs, whereas the complementary condition occurs when the progress of other financial institutions promotes the growth of MFIs. Cull et al. (2014) demonstrated that increasing the number of commercial bank branches per square kilometre helped MFIs to reach out to the impoverished populations. This observation is consistent with the findings of Xiang et al. (2014) who reported that MFIs, particularly in the form of NGOs, have become alternative funding solutions for the poor.

Downscaling refers to when commercial banks target MFI clients. The downscaling of commercial banks makes the continuous operation of Islamic cooperatives (IMFIs) impossible (Husaeni and Dew, 2019). However, several commercial banks have failed in their bids to enter the microfinance market (Bell et al., 2002). An explanation for this failure is the inability of commercial banks to adapt to microfinance needs. However, poverty alleviation can be swiftly accomplished via the collaboration between commercial banks and MFIs to serve the poor.

Bounouala and Rihane (2014) understood this situation and believed that the entry of commercial banks into the microfinance market must be regulated to benefit both parties. The entry technique is determined by the nature of the target market, whether through the development of a microfinance unit, collaboration with an MFI, or credit provision to MFIs. Apart from carefully evaluating how to enter the microfinance market, Messomo Elle (2017) concluded that MFIs require experts and funding sources. If this criterion is met through proper monitoring, the two will grow in sync.

Prior to the emergence of MFIs, most MFI borrowers were financed by moneylenders. However, their emergence is expected to create a more affordable microfinance market with lowered informal interest rates for the poor. According to studies, the expansion of MFIs increases the informal interest rate (Berg et al., 2020; Demont, 2016) and affect borrowers who cannot access MFIs (Venittelli, 2017) since they must continue to rely on moneylenders for services. In some cases, it also demonstrates that moneylenders are still required in the middle of a plethora of MFIs (Arp et al., 2017). The entry of MFIs into the commercial banking sector can impact the efficiency of commercial banks (Abrar et al., 2020). However, other studies argued that MFI entrance into the existing financial industry is negligible (Johnson, 2004).

## 4 Discussion, future research recommendations, and implications

### 4.1 Discussion

Many stakeholders rely on MFIs. MFIs differ from other financial institutions because of their ability to assist the poor while maintaining sustainable financial capacities. However, many people are concerned that MFIs prioritise financial stability over assisting vulnerable populations.

Thus, the issue of mission drifts could taint the performance of the MFIs. Mission drifts are caused by the tendency to emphasise financial performance (Armendáriz et al., 2011; D'Espallier et al., 2010; S. Xu et al., 2016). Mersland and Strøm (2010) and Murti et al. (2018) denied the phenomenon by referring to it as mission development and claiming that there was no evidence of it. Furthermore, the success of MFIs in preserving loan quality and generating a reasonable level of profit encourages other parties, such as commercial banks and capital markets, to route funds to them, and the process is known as commercialisation (Cull et al., 2014; Hamada, 2010). Commercialisation invites a slew of new players, thus shifting the competition level of MFIs (Assefa et al., 2013; de Quidt et al., 2018a).

Various empirical studies on the impact of competition have produced different results (Assefa et al., 2013; Cull et al., 2014; Hossain et al., 2020; Sainz-Fernandez et al., 2018). Nevertheless, it is well known that MFIs cannot evade competition. Competition arises from other MFIs and financial institutions. Furthermore, the poor seek assistance from many sources to progress. The principal goal of MFIs might not be achieved if this assistance is limited. The literature suggests various potential methods to help MFIs address this challenge. MFIs can collaborate to implement better technologies, conduct rigorous screening processes, or obtain other funding options.

### 4.2 Future research recommendations

**Figure 8:**
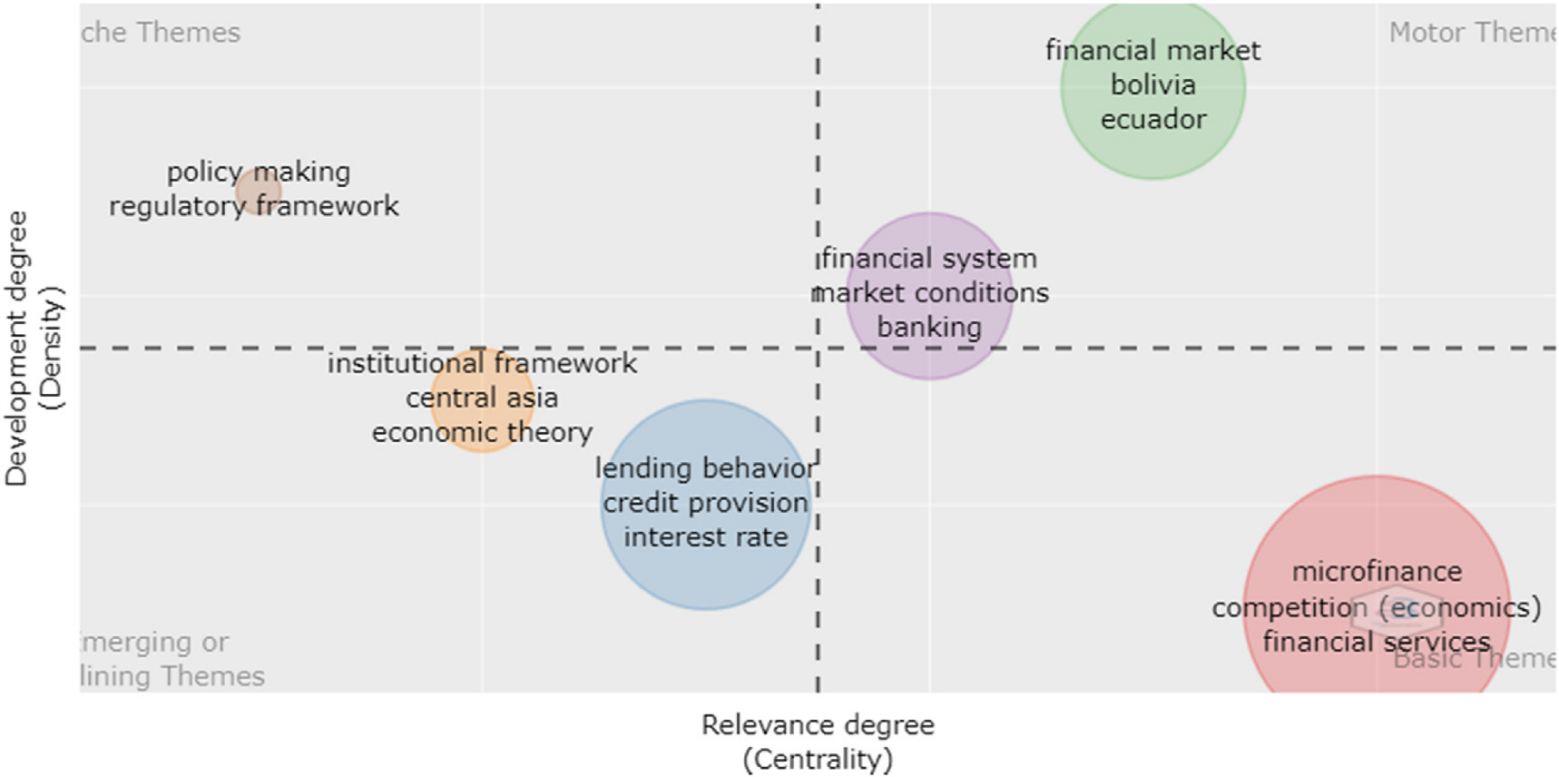
Thematic map of keywords.

To examine future research, the bibliometrix thematic map of keywords was employed (Figure 8). The developed and relevant concepts, also known as the driving themes, are shown in the upper-right quadrant. The lower-right quadrant comprises foundational and transversal features that serve as underlying subjects for discussion with researchers from other fields. The themes in the lower-left quadrant are marginal or vanishing (Della Corte et al., 2019). The upper-left quadrant contains highly specialised topics that have developed strong internal connections but still play a minimal role in the development of competition confronting MFIs (Agbo et al., 2021). The upper-left quadrant depicts the following prospective research: policy-making and regulatory frameworks. Despite these facts, we offered recommendations to regulators on how to foster a suitable competitive environment.

The recommendations for future research on policymaking and regulatory frameworks can be divided into two main categories. First, an adequate framework for regulating the relationship between MFIs and other financial institutions while considering the achievement of the social mission of the latter is required. Further research is proposed to observe the optimal regulations that can facilitate knowledge transfer from commercial banks to MFIs, with a preference for MFIs with a high social reach.

Second, future research can examine the market structures of all kinds of financial institutions in the region to determine the best policy. Additionally, all formal and informal microfinance actors must be represented in these observations. This study largely considered only one element, whether formal or informal. Thus, subsequent ones could incorporate MFIs, commercial banks, and moneylenders.

In addition to being associated with regulation, future research on MFI competition can adopt a Sharia-based approach. To the best of our knowledge, no study has compared the impact of competition on IMFIs or the competition faced by IMFIs and non-IMFIs. As Fianto et al. (2019) stated, many IMFIs are located in rural areas and are confronted by many challenges in their day-to-day operations. Furthermore, the consequences of competition on the MFI types have not been thoroughly discussed. MFIs generally assume different forms. Among them are NGOs, banks, non-bank financial institutions, and cooperatives. The question of which MFI type can endure in the face of competition is one that requires further research.

### 4.3 Implications

Literature review related to the impact of competition has implications for stakeholders. For practitioners, competition impacts MFIs’ internal policy to reduce the negative impact of competition (Cull et al., 2014). The current research results show that competition provides business opportunities for MFIs (Chen and Weiss, 2007) while allowing them to offer lower interest rates (Al-Azzam and Parmeter, 2021). For policy makers, the implication is in the form of policy adjustments that regulate technical matters such as the ideal way for commercial banks to serve micro customers to create synergy between commercial banks and MFIs. In this policy, it is expected that commercial banks can increase their income through service expansion, while on the other hand, MFIs get knowledge transfer in operational management and technology transfer (Bounouala and Rihane, 2014; Messomo Elle, 2017). Nevertheless, those regulations must be implemented carefully since different market structures may impact policy effectiveness (Karimu et al., 2019) especially related to social outreach (de Quidt et al., 2018b).

## 5 Conclusion and research limitations

This study examined the impact of competition on MFI and the prospects for future research, and aids the academia, industry, and policymakers. Based on bibliometric analysis, competition issues were broadly classified into social aspects, performance, market structure, and the existence of other financial institutions. These four perspectives revealed that competition exerts beneficial and adverse effects on MFI. The negative consequences are an increase in non-performing loans, MFIs reduced ability to serve poor consumers, and the inability of MFIs to continue operating.

However, competition would positively impact MFIs if they can adapt to changes in the competition levels. These positive impacts include better portfolio quality, broader client base, and stable profit. Some adaptation strategies include shifting the target market, collaborating with other financial institutions, and improving their operations. These might be embodied in MFIs’ strategies to survive the competition. Concurrently, competition would negatively impact MFIs if they fail to change their internal conditions. This adaptation requires commitment from the management. The study results, which were obtained via bibliometrix, demonstrated that future studies must explore policymaking and regulatory frameworks. It might be achieved by observing the policies that involve all MFI-related sectors, both formal and informal.

The history of MFIs as a financial institution is neither too long nor too short. Academic circles have widely debated MFIs since Muhammad Yunus' Nobel Prize triumph, and the various phenomena that followed it are still being actively discussed. From the start, MFIs were expected to be the answer to the idea of increasing poor people's access to financial aids, but the issues of mission drift, trade-offs, and over-indebtedness that plagued many borrowers cast doubt on the effectiveness of MFIs. Concurrently, the poor continue to require assistance from different parties to evade poverty. Thus, convenient access to affordable services and continued counsel must be prioritised while MFIs gather the resources necessary to accomplish their mission.

This study has some limitations that may need to be overcome in future research. First, this research uses peer-reviewed journals as the only source. Though peer-reviewed journals benefit quality assurance, many studies get published in other forms of publications. This limitation should be considered when interpreting the result. Second, we utilized a selection strategy based on some criteria. Though these criteria have been used in much research, the strategy may not be exhaustive. Different strategies may result in different clusters that can be viewed differently. Consequently, future research should implement different methods to complement this study.

## Declarations

### Author contribution statement

All authors listed have significantly contributed to the development and the writing of this article.

### Funding statement

This work was supported by Universitas Indonesia under the Research Grant contract number NKB-0828/UN2.R3.1/HKP.05.00/2019.

### Data availability statement

Data will be made available on request.

### Declaration of interest's statement

The authors declare no conflict of interest.

### Additional information

No additional information is available for this paper.
